# 1,1′,2,2′-Tetra­methyl-3,3′-(*p*-phenyl­enedimethyl­ene)diimidazol-1-ium bis­(hexa­fluoridophosphate)

**DOI:** 10.1107/S1600536809026336

**Published:** 2009-07-11

**Authors:** Subramaniam Puvaneswary, Yatimah Alias, Seik Weng Ng

**Affiliations:** a Department of Chemistry, University of Malaya, 50603 Kuala Lumpur, Malaysia

## Abstract

The title imidazolium-based ionic-liquid salt, C_18_H_24_N_4_^2+^·2PF_6_^−^, has the cation lying about a center of inversion. The five-membered imidazole ring is disordered over two positions (the methyl substituents are ordered). This imidazole ring is approximately perpendicular to the six-membered phenyl­ene ring [dihedral angle = 81.3 (8)° for one disorder component and 83.8 (8)° for the other; the two components are off-set by 2.7 (8)°]. The crystal is a non-merohedral twin with a twin component of 23%.

## Related literature

For background to imidazolium-based ionic liquid salts, see: Ganesan *et al.* (2008 ▶). For the procedure to manipulate twinned diffraction data, see: Spek (2003). 
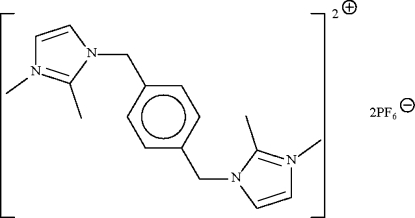


## Experimental

### 

#### Crystal data

C_18_H_24_N_4_^2+^·2PF_6_^−^
*M* *_r_* = 586.35
Triclinic, 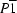

*a* = 7.3808 (3) Å
*b* = 8.2169 (4) Å
*c* = 11.0553 (5) Å
α = 73.435 (3)°
β = 71.173 (3)°
γ = 73.897 (3)°
*V* = 595.43 (5) Å^3^
*Z* = 1
Mo *K*α radiation
μ = 0.29 mm^−1^
*T* = 140 K
0.35 × 0.03 × 0.03 mm

#### Data collection

Bruker SMART APEX diffractometer
Absorption correction: multi-scan (*SADABS*; Sheldrick, 1996 ▶) *T*_min_ = 0.905, *T*_max_ = 0.991
4724 measured reflections
2654 independent reflections
1654 reflections with *I* > 2σ(*I*)
*R*_int_ = 0.037

#### Refinement

*R*[*F* ^2^ > 2σ(*F* ^2^)] = 0.071
*wR*(*F* ^2^) = 0.224
*S* = 1.08
2654 reflections
158 parameters
41 restraints
H-atom parameters constrained
Δρ_max_ = 0.53 e Å^−3^
Δρ_min_ = −0.48 e Å^−3^

### 

Data collection: *APEX2* (Bruker, 2008 ▶); cell refinement: *SAINT* (Bruker, 2008 ▶); data reduction: *SAINT*; program(s) used to solve structure: *SHELXS97* (Sheldrick, 2008 ▶); program(s) used to refine structure: *SHELXL97* (Sheldrick, 2008 ▶); molecular graphics: *X-SEED* (Barbour, 2001 ▶); software used to prepare material for publication: *publCIF* (Westrip, 2009 ▶).

## Supplementary Material

Crystal structure: contains datablocks global, I. DOI: 10.1107/S1600536809026336/tk2496sup1.cif

Structure factors: contains datablocks I. DOI: 10.1107/S1600536809026336/tk2496Isup2.hkl

Additional supplementary materials:  crystallographic information; 3D view; checkCIF report

## Figures and Tables

**Fig. 1.**
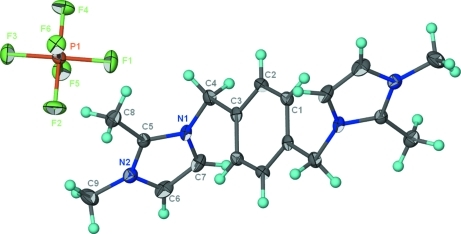
Thermal ellipsoid plot (Barbour, 2001) of [C18H24N4]2+ 2[PF6]- at the 70% probability level; hydrogen atoms are drawn as spheres of arbitrary radius. The imidazolyl ring is disordered; only one component (unprimed) of the disorder is shown. The non-H atoms comprising the asymmetric unit are labelled and the unlabelled atoms are related by 2-x, 2-y, -z.

## supplementary crystallographic information

### Experimental

α,α-Dibromo-*p*-xylene (0.78 g, 3 mmol) and 1,2-dimethylimidazole (0.58 g, 7.6 mmol) were refluxed in DMF (50 ml) for 3 h. The product that separated from solution was collected and washed with ether. Crystals were grown from its solution in water.

The bromide salt (0.46 g, 1 mmol) and sodium hexafluorophosphate (0.17 g, 1 mol) were stirred in water (100 ml) for 24 h. The product that separated from solution was collected and washed with ethanol. Crystals were grown from its solution in DMF.

### Refinement

The imidazolyl ring is disordered over two positions (the two methyl groups are ordered). The ring was refined as a regular pentagon of 1.35 Å sides; the occupancy could not be refined, so the ring was assumed to be disordered in a 1:1 ratio. The *C*–C_methyl_, N–C_methyl_ and *N*–C_methylene_ pairs of distances were restrained to within 0.01 Å of each other. The anisotropic displacement parameters of the primed atoms were restrained to those of the unprimed ones; these were restrained to be nearly isotropic. The two dimethylimidazolyl units were each restrained to be nearly planar.

Carbon-bound H-atoms were placed in calculated positions (C—H 0.95 to 0.99 Å) and were included in the refinement in the riding model approximation, with *U*(H) set to 1.2 to 1.5*U*_eq_(C).

The crystal is a non-merohedral twin; the twin law as given by *PLATON* is (Spek, 2003) (-1 0 0, -0.461 1 - 0.325, 0 0 - 1); the refinement with the inclusion of this gave a twin component of 23%.

### Crystal data

**Table:**

C_18_H_24_N_4_^2+^·2PF_6_^−^	*Z* = 1
*M**_r_* = 586.35	*F*(000) = 298
Triclinic, *P*1	*D*_x_ = 1.635 Mg m^−^^3^
Hall symbol: -P 1	Mo *K*α radiation, λ = 0.71073 Å
*a* = 7.3808 (3) Å	Cell parameters from 951 reflections
*b* = 8.2169 (4) Å	θ = 2.6–23.6°
*c* = 11.0553 (5) Å	µ = 0.29 mm^−^^1^
α = 73.435 (3)°	*T* = 140 K
β = 71.173 (3)°	Prism, colorless
γ = 73.897 (3)°	0.35 × 0.03 × 0.03 mm
*V* = 595.43 (5) Å^3^	

### Data collection

**Table:**

Bruker SMART APEX diffractometer	2654 independent reflections
Radiation source: fine-focus sealed tube	1654 reflections with *I* > 2σ(*I*)
graphite	*R*_int_ = 0.037
ω scans	θ_max_ = 27.5°, θ_min_ = 2.0°
Absorption correction: multi-scan (*SADABS*; Sheldrick, 1996)	*h* = −9→9
*T*_min_ = 0.905, *T*_max_ = 0.991	*k* = −10→10
4724 measured reflections	*l* = −14→14

### Refinement

**Table:**

Refinement on *F*^2^	Primary atom site location: structure-invariant direct methods
Least-squares matrix: full	Secondary atom site location: difference Fourier map
*R*[*F*^2^ > 2σ(*F*^2^)] = 0.071	Hydrogen site location: inferred from neighbouring sites
*wR*(*F*^2^) = 0.224	H-atom parameters constrained
*S* = 1.08	*w* = 1/[σ^2^(*F*_o_^2^) + (0.0937*P*)^2^ + 1.0627*P*] where *P* = (*F*_o_^2^ + 2*F*_c_^2^)/3
2654 reflections	(Δ/σ)_max_ = 0.001
158 parameters	Δρ_max_ = 0.53 e Å^−^^3^
41 restraints	Δρ_min_ = −0.48 e Å^−^^3^

### Fractional atomic coordinates and isotropic or equivalent isotropic displacement parameters (Å^2^)

**Table:**

	*x*	*y*	*z*	*U*_iso_*/*U*_eq_	Occ. (<1)
P1	0.27195 (18)	0.30311 (17)	0.76433 (12)	0.0235 (3)	
F6	0.2767 (5)	0.4311 (4)	0.6246 (3)	0.0364 (8)	
F4	0.3686 (5)	0.1439 (4)	0.6946 (3)	0.0428 (8)	
F5	0.2653 (5)	0.1760 (4)	0.9036 (3)	0.0400 (8)	
F3	0.0593 (4)	0.2794 (5)	0.7741 (3)	0.0417 (8)	
F2	0.1736 (5)	0.4638 (4)	0.8340 (3)	0.0494 (10)	
F1	0.4829 (4)	0.3294 (5)	0.7547 (3)	0.0440 (9)	
C1	1.1492 (7)	1.0037 (6)	0.3848 (4)	0.0233 (10)	
H1	1.2518	1.0052	0.3063	0.028*	
C2	1.0666 (7)	0.8581 (7)	0.4421 (5)	0.0259 (10)	
H2	1.1122	0.7612	0.4018	0.031*	
C3	0.9177 (6)	0.8525 (6)	0.5581 (5)	0.0218 (10)	
C4	0.8353 (7)	0.6892 (6)	0.6166 (5)	0.0263 (11)	
H4A	0.7815	0.6670	0.5530	0.032*	0.50
H4B	0.9412	0.5891	0.6352	0.032*	0.50
H4C	0.7946	0.6625	0.5486	0.032*	0.50
H4D	0.9413	0.5921	0.6398	0.032*	0.50
N1	0.683 (2)	0.706 (4)	0.7365 (15)	0.0231 (17)	0.50
C5	0.493 (3)	0.753 (2)	0.7349 (14)	0.0216 (14)	0.50
N2	0.3833 (7)	0.7643 (18)	0.858 (2)	0.0250 (13)	0.50
C6	0.505 (3)	0.724 (4)	0.9349 (6)	0.034 (2)	0.50
H6	0.4677	0.7219	1.0259	0.040*	0.50
C7	0.690 (2)	0.688 (5)	0.860 (2)	0.031 (3)	0.50
H7	0.8057	0.6562	0.8892	0.038*	0.50
N1'	0.670 (2)	0.695 (4)	0.7318 (14)	0.0231 (17)	0.50
C5'	0.477 (3)	0.749 (2)	0.7430 (16)	0.0216 (14)	0.50
N2'	0.3852 (8)	0.7552 (19)	0.869 (2)	0.0250 (13)	0.50
C6'	0.523 (3)	0.705 (4)	0.9363 (7)	0.034 (2)	0.50
H6'	0.4993	0.6975	1.0272	0.040*	0.50
C7'	0.6988 (18)	0.668 (5)	0.851 (2)	0.031 (3)	0.50
H7'	0.8213	0.6300	0.8718	0.038*	0.50
C8	0.3963 (5)	0.7903 (4)	0.6307 (4)	0.0303 (11)	
H8A	0.4926	0.7588	0.5517	0.046*	0.50
H8B	0.3380	0.9143	0.6118	0.046*	0.50
H8C	0.2937	0.7228	0.6587	0.046*	0.50
H8D	0.5010	0.7607	0.5544	0.046*	0.50
H8E	0.3388	0.9145	0.6118	0.046*	0.50
H8F	0.2953	0.7233	0.6506	0.046*	0.50
C9	0.1775 (5)	0.8077 (4)	0.9104 (4)	0.0407 (14)	
H9A	0.1131	0.8479	0.8392	0.061*	0.50
H9B	0.1463	0.9001	0.9582	0.061*	0.50
H9C	0.1311	0.7052	0.9703	0.061*	0.50
H9D	0.1199	0.7958	0.8458	0.061*	0.50
H9E	0.1428	0.9291	0.9183	0.061*	0.50
H9F	0.1271	0.7344	0.9955	0.061*	0.50

### Atomic displacement parameters (Å^2^)

**Table:**

	*U*^11^	*U*^22^	*U*^33^	*U*^12^	*U*^13^	*U*^23^
P1	0.0225 (6)	0.0259 (7)	0.0221 (6)	−0.0038 (5)	−0.0050 (5)	−0.0074 (5)
F6	0.0438 (18)	0.0321 (17)	0.0305 (16)	−0.0089 (14)	−0.0136 (14)	0.0030 (13)
F4	0.051 (2)	0.0294 (17)	0.0420 (19)	0.0005 (14)	−0.0048 (15)	−0.0157 (15)
F5	0.0462 (19)	0.046 (2)	0.0278 (16)	−0.0157 (15)	−0.0126 (14)	0.0012 (14)
F3	0.0283 (17)	0.060 (2)	0.0410 (19)	−0.0154 (15)	−0.0101 (14)	−0.0102 (16)
F2	0.056 (2)	0.044 (2)	0.050 (2)	−0.0074 (17)	−0.0001 (17)	−0.0294 (17)
F1	0.0302 (17)	0.064 (2)	0.0415 (19)	−0.0187 (16)	−0.0123 (14)	−0.0048 (17)
C1	0.020 (2)	0.030 (3)	0.019 (2)	−0.0075 (19)	−0.0014 (17)	−0.0062 (19)
C2	0.022 (2)	0.027 (3)	0.028 (3)	−0.0026 (19)	−0.0044 (19)	−0.010 (2)
C3	0.017 (2)	0.024 (2)	0.025 (2)	0.0003 (18)	−0.0121 (18)	−0.0026 (19)
C4	0.020 (2)	0.027 (3)	0.031 (3)	−0.005 (2)	−0.0047 (19)	−0.006 (2)
N1	0.020 (2)	0.026 (3)	0.026 (2)	−0.010 (2)	−0.0078 (19)	−0.0027 (18)
C5	0.022 (3)	0.022 (2)	0.021 (3)	−0.009 (2)	−0.003 (2)	−0.0026 (19)
N2	0.027 (2)	0.025 (2)	0.024 (3)	−0.0109 (17)	−0.0049 (17)	−0.003 (2)
C6	0.046 (4)	0.042 (5)	0.017 (2)	−0.019 (4)	−0.010 (2)	−0.001 (2)
C7	0.035 (3)	0.036 (7)	0.032 (4)	−0.014 (3)	−0.017 (3)	−0.003 (3)
N1'	0.020 (2)	0.026 (3)	0.026 (2)	−0.010 (2)	−0.0078 (19)	−0.0027 (18)
C5'	0.022 (3)	0.022 (2)	0.021 (3)	−0.009 (2)	−0.003 (2)	−0.0026 (19)
N2'	0.027 (2)	0.025 (2)	0.024 (3)	−0.0109 (17)	−0.0049 (17)	−0.003 (2)
C6'	0.046 (4)	0.042 (5)	0.017 (2)	−0.019 (4)	−0.010 (2)	−0.001 (2)
C7'	0.035 (3)	0.036 (7)	0.032 (4)	−0.014 (3)	−0.017 (3)	−0.003 (3)
C8	0.033 (3)	0.027 (3)	0.034 (3)	−0.006 (2)	−0.015 (2)	−0.004 (2)
C9	0.032 (3)	0.042 (3)	0.041 (3)	−0.011 (2)	0.008 (2)	−0.016 (3)

### Geometric parameters (Å, °)

**Table:**

P1—F4	1.586 (3)	C6—C7	1.3500
P1—F5	1.590 (3)	C6—H6	0.9500
P1—F1	1.597 (3)	C7—H7	0.9500
P1—F2	1.597 (3)	N1'—C5'	1.3500
P1—F6	1.598 (3)	N1'—C7'	1.3500
P1—F3	1.600 (3)	C5'—N2'	1.3500
C1—C2	1.388 (7)	C5'—C8	1.459 (7)
C1—C3^i^	1.393 (6)	N2'—C6'	1.3500
C1—H1	0.9500	N2'—C9	1.428 (6)
C2—C3	1.392 (6)	C6'—C7'	1.3500
C2—H2	0.9500	C6'—H6'	0.9500
C3—C1^i^	1.393 (6)	C7'—H7'	0.9500
C3—C4	1.514 (7)	C8—H8A	0.9800
C4—N1	1.449 (7)	C8—H8B	0.9800
C4—N1'	1.452 (7)	C8—H8C	0.9800
C4—H4A	0.9900	C8—H8D	0.9800
C4—H4B	0.9900	C8—H8E	0.9800
C4—H4C	0.9900	C8—H8F	0.9800
C4—H4D	0.9900	C9—H9A	0.9800
N1—C5	1.3500	C9—H9B	0.9800
N1—C7	1.3500	C9—H9C	0.9800
C5—N2	1.3500	C9—H9D	0.9800
C5—C8	1.462 (7)	C9—H9E	0.9800
N2—C6	1.3500	C9—H9F	0.9800
N2—C9	1.422 (6)		
			
F4—P1—F5	90.52 (18)	C5'—N1'—C4	131 (2)
F4—P1—F1	90.71 (19)	C7'—N1'—C4	121 (2)
F5—P1—F1	90.42 (18)	N1'—C5'—N2'	108.0
F4—P1—F2	179.6 (2)	N1'—C5'—C8	122 (2)
F5—P1—F2	89.74 (19)	N2'—C5'—C8	130 (2)
F1—P1—F2	89.58 (19)	C6'—N2'—C5'	108.0
F4—P1—F6	89.62 (18)	C6'—N2'—C9	132 (2)
F5—P1—F6	179.52 (18)	C5'—N2'—C9	120 (2)
F1—P1—F6	90.04 (18)	N2'—C6'—C7'	108.0
F2—P1—F6	90.11 (18)	N2'—C6'—H6'	126.0
F4—P1—F3	89.98 (19)	C7'—C6'—H6'	126.0
F5—P1—F3	89.90 (18)	C6'—C7'—N1'	108.0
F1—P1—F3	179.2 (2)	C6'—C7'—H7'	126.0
F2—P1—F3	89.73 (19)	N1'—C7'—H7'	126.0
F6—P1—F3	89.64 (17)	C5'—C8—H8A	113.7
C2—C1—C3^i^	120.3 (4)	C5—C8—H8A	109.5
C2—C1—H1	119.8	C5'—C8—H8B	109.7
C3^i^—C1—H1	119.8	C5—C8—H8B	109.5
C1—C2—C3	120.7 (4)	H8A—C8—H8B	109.5
C1—C2—H2	119.7	C5'—C8—H8C	104.9
C3—C2—H2	119.7	C5—C8—H8C	109.5
C2—C3—C1^i^	119.0 (4)	H8A—C8—H8C	109.5
C2—C3—C4	118.2 (4)	H8B—C8—H8C	109.5
C1^i^—C3—C4	122.8 (4)	C5'—C8—H8D	109.5
N1—C4—C3	110.7 (13)	C5—C8—H8D	105.1
N1'—C4—C3	115.8 (13)	C5'—C8—H8E	109.5
N1—C4—H4A	109.5	C5—C8—H8E	109.2
N1'—C4—H4A	103.2	H8A—C8—H8E	109.4
C3—C4—H4A	109.5	H8C—C8—H8E	109.9
N1—C4—H4B	109.5	H8D—C8—H8E	109.5
N1'—C4—H4B	110.3	C5'—C8—H8F	109.5
C3—C4—H4B	109.5	C5—C8—H8F	114.0
H4A—C4—H4B	108.1	H8A—C8—H8F	105.3
N1—C4—H4C	114.7	H8B—C8—H8F	109.1
N1'—C4—H4C	108.3	H8D—C8—H8F	109.5
C3—C4—H4C	108.3	H8E—C8—H8F	109.5
N1—C4—H4D	107.2	N2—C9—H9A	109.5
N1'—C4—H4D	108.3	N2'—C9—H9A	114.6
C3—C4—H4D	108.3	N2—C9—H9B	109.5
C5—N1—C7	108.0	N2'—C9—H9B	108.2
C5—N1—C4	120 (2)	H9A—C9—H9B	109.5
C7—N1—C4	132 (2)	N2—C9—H9C	109.5
N2—C5—N1	108.0	N2'—C9—H9C	105.5
N2—C5—C8	119 (2)	H9A—C9—H9C	109.5
N1—C5—C8	133 (2)	H9B—C9—H9C	109.5
C6—N2—C5	108.0	N2—C9—H9D	105.5
C6—N2—C9	121 (2)	N2'—C9—H9D	109.5
C5—N2—C9	131 (2)	N2—C9—H9E	108.2
N2—C6—C7	108.0	N2'—C9—H9E	109.5
N2—C6—H6	126.0	H9D—C9—H9E	109.5
C7—C6—H6	126.0	N2—C9—H9F	114.6
C6—C7—N1	108.0	N2'—C9—H9F	109.5
C6—C7—H7	126.0	H9D—C9—H9F	109.5
N1—C7—H7	126.0	H9E—C9—H9F	109.5
C5'—N1'—C7'	108.0		
			
C3^i^—C1—C2—C3	−0.8 (8)	C9—N2—C6—C7	179.8 (3)
C1—C2—C3—C1^i^	0.8 (8)	N2—C6—C7—N1	0.0
C1—C2—C3—C4	−179.3 (4)	C5—N1—C7—C6	0.0
C2—C3—C4—N1	180.0 (11)	C4—N1—C7—C6	178 (2)
C1^i^—C3—C4—N1	−0.1 (12)	C3—C4—N1'—C5'	87.8 (19)
C2—C3—C4—N1'	−175.4 (11)	C3—C4—N1'—C7'	−81.7 (9)
C1^i^—C3—C4—N1'	4.5 (12)	C7'—N1'—C5'—N2'	0.0
N1'—C4—N1—C5	−44 (19)	C4—N1'—C5'—N2'	−170.5 (19)
C3—C4—N1—C5	97.1 (15)	C7'—N1'—C5'—C8	179.9 (3)
N1'—C4—N1—C7	139 (21)	C4—N1'—C5'—C8	9.4 (19)
C3—C4—N1—C7	−80.4 (10)	N1'—C5'—N2'—C6'	0.0
C7—N1—C5—N2	0.0	C8—C5'—N2'—C6'	−179.9 (3)
C4—N1—C5—N2	−178.0 (17)	N1'—C5'—N2'—C9	−179.8 (3)
C7—N1—C5—C8	179.9 (3)	C8—C5'—N2'—C9	0.3 (4)
C4—N1—C5—C8	1.9 (17)	C5'—N2'—C6'—C7'	0.0
N1—C5—N2—C6	0.0	C9—N2'—C6'—C7'	179.8 (3)
C8—C5—N2—C6	−179.9 (2)	N2'—C6'—C7'—N1'	0.0
N1—C5—N2—C9	−179.8 (3)	C5'—N1'—C7'—C6'	0.0
C8—C5—N2—C9	0.2 (4)	C4—N1'—C7'—C6'	171.6 (18)
C5—N2—C6—C7	0.0		

Symmetry codes: (i) −*x*+2, −*y*+2, −*z*+1.
